# NewTools: a novel collaboration across the Norwegian food system with the aim of developing tools for food system transformation

**DOI:** 10.1017/S1368980025100785

**Published:** 2025-08-05

**Authors:** Helle Margrete Meltzer, Kaja Lund-Iversen, Arnfinn Helleve, Marianne Hope Abel, Anne Lene Løvhaug, Marianne S. Morseth, Hanne Fjerdingby Olsen, Trond Arild Ydersbond, Knut-Inge Klepp

**Affiliations:** 1 Centre for Sustainable Diets, Norwegian Institute of Public Health, Oslo, Norway; 2 Department of Research Administrative Support, Norwegian Institute of Public Health, Oslo, Norway; 3 Centre for Evaluation of Public Health Measures, Norwegian Institute of Public Health, Oslo, Norway; 4 Department of Physical Health and Ageing, Norwegian Institute of Public Health, Oslo, Norway; 5 Department of Nursing and Health Promotion, Oslo Metropolitan University, Oslo, Norway; 6 Department of Animal and Aquacultural Sciences, Norwegian University of Life Sciences, Ås, Norway; 7 Department of IT, Statistics Norway, Oslo, Norway

**Keywords:** Conceptual paper, Food system, Sustainability, Norway, Sustainability score, Nutrient profiling, Multidisciplinary project, Partner engagement

## Abstract

**Objective:**

The NewTools project aims to support the transformation of the food system by developing summary scores for the nutritional value and environmental and social sustainability of foods and exploring potential applications. In this conceptual paper, we present the governance, objectives, conceptualisation and expected outcomes of the NewTools project.

**Design:**

A cross-sector research partnership involving actors across the Norwegian food system.

**Setting:**

The need to transform food systems both globally, regionally and nationally.

**Participants:**

A broad constellation of twenty-eight project partners includes research institutions, governmental agencies, food industry and Non-governmental organization (NGO).

**Expected results:**

Outputs from the project will include the development and testing of a score for nutritional quality using the European Nutri-Score version 2023 as a starting point, identifying of indicators to measure social and environmental sustainability, proposing weighting of these into one or several summary scores, pilots testing potential applications of use for the scores and protocols for relevant spin-off projects.

**Conclusion:**

The multitude of perspectives represented by this unique variety of partners is seen as valuable to better understand the opportunities and limitations of the proposed tools designed to foster transformations towards a more resilient and sustainable food system.

Food systems are at the centre of a poly crisis; the world is exceeding several planetary boundaries, and food production is a significant contributor to human impact on both climate and nature. Furthermore, unhealthy diets are the primary driver of obesity and preventable diet-related diseases. All income groups fail to meet dietary recommendations. Today’s diet in most countries is neither sustainable from a health, environmental nor social perspective^(1)^. Addressing these challenges requires action and involvement from all sectors in society.

One of many approaches to handling the crisis is to acquire and communicate information about the environmental, social and economic footprint of our foods. Such information can be used to rank foods, for example through a scoring system/food profiling model that assesses the nutritional and sustainability values of foods and beverages. Food scores can potentially be used in various ways, such as for front-of-pack labelling, public food procurement, public food provisions, marketing regulation and health taxation. Scoring systems can also be used by the food industry to reformulate products and can serve as monitoring tools for evaluating initiatives and policies across several sectors of the food system^(2)^.

In Europe, several food scoring systems for nutritional quality underpin different nutrition policy measures based on different principles and development processes. In Norway, a profiling system with defined nutrition criteria for thirty-three food groups underpins the Nordic Keyhole, an endorsement logo that can be used to indicate healthier options within each food group^(3)^. The development was led by Nordic government health authorities in consultation with the food industry. Another scoring system, which applies criteria across four major food categories, underpins the French Nutri-Score, a front of pack nutrition label that provides a five-level summary score for nutrition quality, hence indicating both healthier and less healthy foods^(4)^. The algorithm was developed by an independent research group^(4)^, which is in line with The WHO who recommends that nutrition profiling systems are developed by independent research groups to ensure scientific integrity^(5)^. Nutri-Score has been implemented by governments in countries covering more than half of the EU population. Other countries are, however, reluctant to implement the labelling scheme due to its reductionistic approach to food quality, inconsistencies in how it discriminates between healthy and less healthy food products and due to poorer scores being assigned to many national and traditional foods such as cured meat and cheese products^(6–8)^.

Developing a scoring system for the sustainability of foods and beverages is inherently a complex task, as it must take into account production processes and not merely the final product. Such work has come far shorter than the work on scoring systems for nutritional quality. Depending on how sustainability is defined, evaluating food sustainability can involve aspects of environmental, social and economic sustainability. This holistic approach is gradually developing from the initial focus on solely mitigating greenhouse gas emissions and is in line with the sustainability strategies of the European Commission^(9)^. Currently, there is no universally accepted methodology for documenting the sustainability of food products, although there are some proposals. The Product Environmental Footprint (PEF) is a multi-criteria measure of the environmental performance of a product or service throughout its life cycle. Within the EU, PEF category rules, which provides detailed regulations and guidelines for specific product categories, have been developed for some food groups, such as beer, dairy and pasta^(10–12)^ and category rules for marine fish for human consumption are about to be published (April 2025)^(13)^. The PEF development involves a working group process with representation from industry and other societal stakeholders. PEF is based on life cycle assessment, which is a widely used tool for documenting environmental impacts throughout a product’s or service’s life cycle^(14)^. A similar but less well-known method for mapping social sustainability is social life cycle assessment (S-life cycle assessment), with guidelines developed by the UN Environment Program^(15)^.

Scoring systems for environmental impact of foods have materialised into labelling schemes in some places, such as the Eco-Score, which was introduced in France through a private sector initiative^(16)^. With the new EU Directive on Empowering Consumers for the Green Transition to be implemented in 2025, unverified and non-certified environmental claims made by private actors will be prohibited^(17)^, indicating the need for authorities to develop standardised methodology and certified labelling schemes.

Despite these methodological and policy challenges, scoring systems assessing the nutritional quality and sustainability of foods can potentially lead to food systems change through influencing food value chains, food environments, consumer behaviour and diets and ultimately dietary, health, environmental and social outcomes^(18)^. Due to their potential applicability as innovation, scientific and policy tools, it is important to inform the development of scoring systems with perspectives from different food systems perspectives. The Norwegian NewTools project is an example of a collaborative effort to mobilise a diverse set of food system actors to address the challenges connected to establishing summary scores for the nutritional, environmental and social dimensions of foods.

In this conceptual paper,^(19)^ we present the governance, objectives, conceptualisation and expected outcomes of the NewTools project.

## Funding, governance and partners

The NewTools project has received funding from the Research Council of Norway and their program on ‘Collaborative Projects to meet Societal and Industry-related Challenges’^(20)^. The goal of this program is to develop new knowledge and generate the research competence needed across societal sectors to address important societal challenges. Funded projects are required to include the participation of Norwegian collaboration partners who are not research organisations but rather are civil society, public and private actors who will ensure that issues, research activities and results become relevant and beneficial to society. Furthermore, it is a requirement that non-research partners contribute with a minimum of 10% of the overall budget of the project.

A unique feature of the NewTools is therefore the involvement of Norwegian food system actors across the food chain, including representatives of primary producers, manufacturers, retailers, consumers, government agencies, civil society and academia. In total, the project involves twenty-eight partners (Table 1). They were invited based on their involvement with, and knowledge of, the Norwegian food system, many of whom had been involved in an earlier project, Matdugnaden, where mapping of the Norwegian food system was a main aim^(21)^.

**Table 1.** NewTools partner institutions, their financial category and the main expertise they contribute with in the project

Partners	Role	Expertise relevant to the project, key-words
Research institutions		
Norwegian Institute for Sustainability Research	1	Life cycle assessment, environmental and social impacts
Norwegian Institute of Public Health	1	Public health research, nutrient profiling of food
Norwegian University of Life Sciences	1	Sustainable food production systems, animal and plant sciences
Oslo Metropolitan University	1	Public health nutrition research, consumer perspectives
Statistics Norway	1	IT, portal, statistics
University of Oslo	1	Food consumption research, environmental indicators
Institute of Marine Research	3	Seafood nutrition and environment research
Food industry		
Animalia AS	2	Umbrella organisation, meat, and egg sector
Bama Group	2	Fruit and vegetable import and manufacturing
Felleskjøpet Agri SA	2	Cooperative, grain, and fodder sector
Norgesmøllene	2	Grain and flour manufacturer
NORTURA	2	Cooperative, meat and egg producer and manufacturer
TINE SA	2	Cooperative, milk and dairy producer and manufacturer
COOP Norge SA	3	Retailer and food manufacturer
FoodDrinkNorway^*^	3	Member organization representing the food and drink industry in Norway
NorgesGruppen	3	Retailer and food manufacturer
Norwegian Farmers and Smallholders Union^*^	3	Farmer and small-holder organisation
Orkla Foods Norway	3	Food manufacturer, multiple brands
REMA 1000	3	Retailer and food manufacturer
Norwegian Seafood Federation^*^	3	Umbrella organisation, seafood industry companies including aquaculture, technology providers and veterinary service companies
Governmental agencies		
Norwegian Agricultural Agency	3	Public agency on agriculture and forest
Norwegian Directorate of Health^*^	3	Public agency diet and public health
Norwegian Food Safety Authority	3	Public agency on food safety and food regulation
Civil society organisations		
Norwegian Consumer Council	1	Consumer perspective and interests
EAT	3	International and global food system perspectives
Future in Our Hands	3	Environmental issues
Debio – The Food Choice	3	Organic food procurement and cantinas
Spire – youth organisation	3	Environmental issues, biodiversity, food security, equality for sustainable development

RCN, Research Council of Norway.

* Members of the NewTools project strategic advisory board, together with the independent actor Rethink Food.

1) Partners receiving financial support from RCN.

2) Partners nor receiving financial support from RCN, but who support the total budget through in-kind work and have to report this to RCN.

3) Partners covering their own contributions and not obliged to report to RCN.

NewTools has three categories of partners: (1) Partners receiving financial support from RCN, (2) partners not receiving financial support from RCN, but who support the total budget through in-kind work and have to report this to RCN and (3) partners covering their own contributions and not obliged to report to RCN. The grant from the funder is allocated to six research institutions and one civil society organisation. No monetary support is provided by the food industry partners to the participating research institutions.

The project has two advisory boards that are involved in strategic and scientific issues, respectively, with representatives from five national and international organisations. Interested non-partners are also invited to attend public project meetings and to follow the project through the NewTools project webpage (https://www.fhi.no/kl/studier/newtools/) and newsletters.

## Conceptualisation of the project

**Figure 1.**
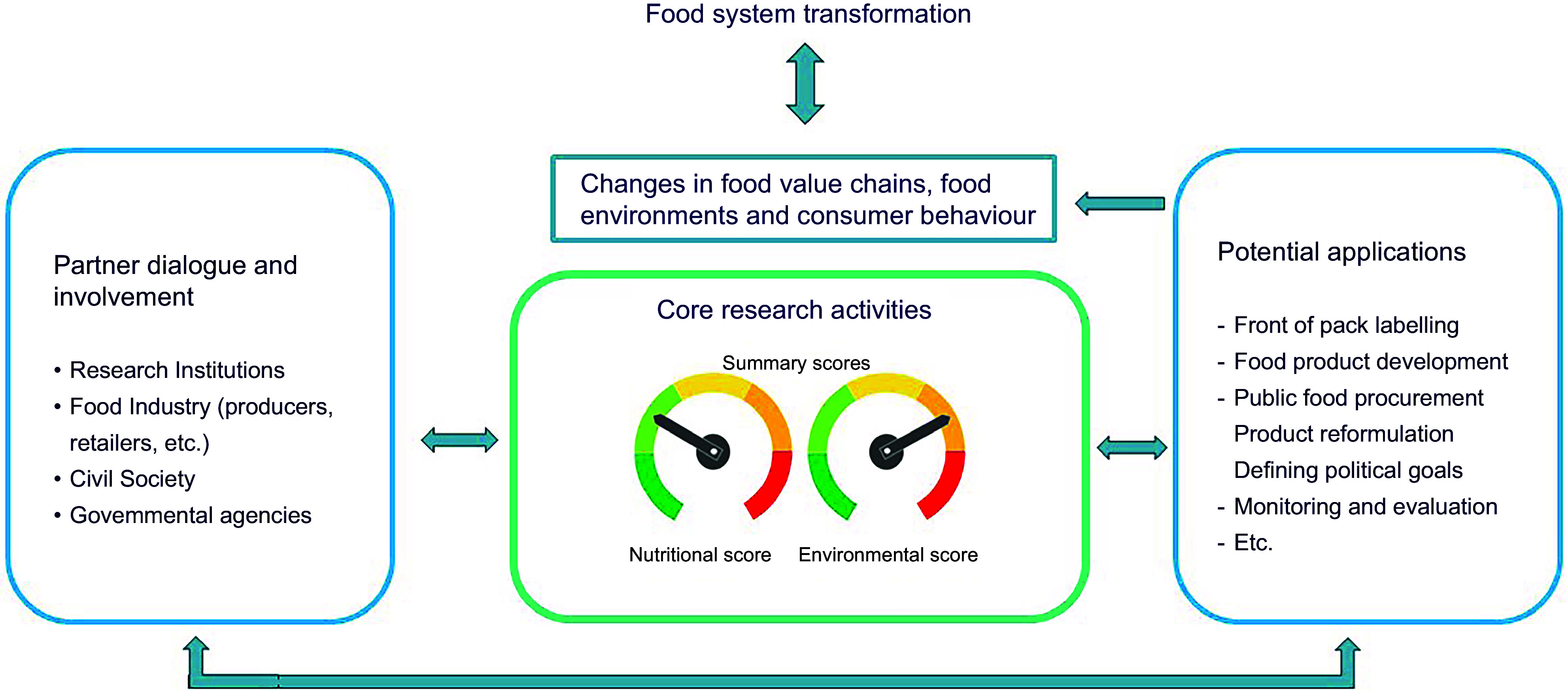
Conceptual model of the NewTools project.

The overall concept of the project is illustrated in Figure 1. A range of scientific disciplines, spanning from food science and nutrition to agricultural production, life cycle analysis, environmental footprint and sustainability research, social science, statistics and public health, are necessary for the successful implementation of the project. This will help build a strong interdisciplinary competence network across research institutions and between researchers. The researchers are responsible for defining the indicators and scoring system based on scientific evidence.

Actors representing different part of the Norwegian food system are also actively involved in the project. Project partners representing government agencies, government agencies and Non-governmental organization (NGO) will provide perspectives from beyond the research spheres, for example on which relevant indicators to include, on feasibility of use and of potential unexpected consequences of indicators, methods or scores. Achieving insights from the involved actors to the issues the project aims to solve will provide a more holistic and systems-based development of solutions. Researchers will therefore consider input from the partners in the development of scoring systems. Partner involvement is described further below.

The project may contribute to food systems change if the project outcomes – the scoring systems – are applied and implemented by food systems actors. This will require research outcomes that are feasible to use for a range of actors and that are also aligned with existing policy and regulatory frameworks.

### Partner involvement in NewTools

The project consortium includes partners representing various parts of the food system, all with different capacities, resources, and interests.

To facilitate partner involvement, secure transparency, prevent and manage potential conflicts and secure research integrity, the project has developed a framework for engagement^(22)^. The developmental process of the framework has been guided by recommendations for methodological framework development,^(23)^ and the content of the framework has been based on the WHO’ Framework for Engagement of Non-State Actors^(24)^.

The framework has been developed through an iterative process between researchers and partners, and its development will be outlined in a separate article. The framework outlines the roles and responsibilities of the different partners and specifies that the responsibility for decision-making processes is placed on research partners. To facilitate dialogue and involvement, the framework also defines concrete forms of interaction, such as dialogue-based workshops, webinars, meetings, invitations to provide written feedback to drafts and compiled research and involvement in developing pilots and testing the scoring systems in specific contexts.

However, involvement of such a diverse range of partners also involves risks. Public health literature points to the potential that conflicts of interest may undermine research processes and outcomes to the benefit of commercial interests at the expense of public health (Gilmore 2023),^(25)^ and the potential of tension between partners representing different values, interests and power is high (Fanzo *et al.* 2021)^(26)^. The project therefore has to balance the involvement of partners, which intends to benefit the project, while safeguarding research integrity.

The following ethical questions and issues related to the scientific independence of the research findings are foreseen, and mitigation strategies developed accordingly:

- Conflict of interest: This may bias the integrity of the research and its outcomes^(27,28)^. Researchers may be influenced, consciously or unconsciously, to produce results that favour their partners, such as governmental institutions, NGO or the business interests of their corporate partners, compromising the objectivity of their research. NewTools project framework for collaboration aims to secure transparency and thereby handle such issues^(22)^.
- Intellectual property and access to knowledge: There may be restrictions on data access and sharing, for example, owing to commercial interests. One approach to mitigating this could be to approximate versions of sensitive data (e.g. food or fodder recipes). In this way, results may be verified independently, and other researchers will be able to build upon the research. It is the responsibility of the researchers and project partners involved to find solutions to the use and exchange of such data within the project and within acceptable security regimes.
- Accountability and transparency within the project: Ensuring that there are adequate mechanisms for accountability and oversight is critical for maintaining ethical standards in collaboration. Clear communication to the consortium from the coordinator, information sharing and openness in all processes, real involvement of partners, easy access to research results and regular consortium meetings to openly discuss project aims and progress are measures employed in this respect.
- Public trust and perception: Collaborations that appear to compromise scientific integrity can erode public trust in both the scientific community and businesses involved. Accordingly, the NewTools project emphasises transparency and adequate documentation of all its activities.

Given the multi-actor and cross-disciplinary character of this research project, it is of scientific interest to investigate the partners’ expectations and experiences of being involved. There is little empirical evidence on how cross-sector collaboration functions in concrete partnerships, and NewTools aims to provide such evidence^(26,29,30)^. Two rounds of interviews with project partners will be conducted, aiming to contribute to understanding of factors that facilitate and challenge collaborations between diverse participants in cross-sector partnerships.

The objectives and research questions in the NewTools project

**Specific objectives include:**

- Establish a framework for engagement to facilitate involvement of project partners while safeguarding research integrity
- Develop a summary score for nutritional quality by refining existing scores and evaluating its performance from a Nordic perspective
- Assess relevant indicators for combining environmental and social sustainability into one or two summary scores
- Establish a platform for sharing relevant data, methods, and information, facilitating cooperative workflows and collaboration among food system partners
- Identify, plan and test application of NewTools scoring at relevant entry points in the Norwegian food system

**To address these objectives, several research questions have been formulated:**

- What are the different food system actors’ expectations, experiences and evaluation of participation in a cross-sector research partnership?
- From a Nordic perspective, what are the shortcomings and potential improvements to the scoring systems currently being developed in other countries, especially in Europe, on nutrition quality and sustainability?
- Which indicators are the most relevant if summary scores on environmental and social sustainability are to be generated, what are their relative importance, and is it feasible to combine them into a single sustainability score?
- How valid and reliable are the proposed summary scores, that is, how do the two (or more) scoring systems discriminate more healthy foods from less healthy and more sustainable foods from less sustainable, applying different metrics for health and sustainability?
- At the food system level, how may the results from NewTools be used to guide decisions at entry points identified by Norwegian food system actors, such as food producers, food retailers, policy makers and consumers, nationally and internationally?
- What are the potential action points for policymaking within the food systems emerging from this co-creation process – for Norway, Nordic countries and the EU?

## Expected outcomes

### Develop and evaluate a summary score for the nutritional quality of foods

The use of nutritional summary scores has the potential to improve diet quality and decrease mortality from diet-related non-communicable diseases^(31)^. In NewTools, we will build on the scientific literature regarding existing nutritional scores and their strengths and weaknesses. The French Nutri-Score will be selected as a starting point as it has been developed by researchers, tested and shown to be predictive of healthier food choices and is already in use in Europe^(32)^. First, we aim to evaluate the revised Nutri-Score from a Norwegian perspective, that is, to assess how it will perform in a Norwegian setting, including the identification of potential strengths and weaknesses in ranking foods from a health perspective. This includes calculating the score in a Norwegian food database and evaluating discriminatory ability and alignment with Norwegian food-based dietary guidelines^(33)^ and with nutrition experts’ ranking of foods by healthiness^(34)^. Also, we aim to explore food-system actors’ perspectives on the scoring^(35)^. This comprehensive evaluation will provide in-depth insight into the potential challenges of the Nutri-Score algorithms in a Norwegian context. Through testing and examination of adjustments of the scoring, revisions of the Nutri-Score can be proposed^(36)^. In line with policy recommendations for development of food profiling models^(5)^, non-research partners with vested interests will not be directly involved in the development process except for identifying strengths and challenges of the score. This is to ensure that the score development will not been influenced by conflicts of interests. To further investigate the usefulness, application of the nutrient profiling system to dietary data and studying associations to relevant health outcomes will be conducted.

### Develop scores for the environmental and social sustainability of foods

As already mentioned, we aim to develop a framework for quantifying the sustainability of food products, including both environmental (e.g. climate, eutrophication and land use) and social aspects (e.g. occupational accidents, fair salary and animal welfare). The current standardised methodology (European PEF;^(37)^), guidelines for S-life cycle assessment^(38)^, international scientific literature and knowledge-building research projects that have developed a basis for sustainability assessments will be the basis for development of the scoring systems for environmental and social sustainability. Thus, the most important environmental and social impact categories from food products will be selected along with a relevant set of indicators^(39)^. Existing frameworks covering both environmental and social sustainability will be tested for usability on product level to identify possible gaps related to the defined impact categories^(40)^. In addition, we will provide an overview of the available data of sufficient quality to measure the indicators. Statistical analyses will also be carried out to find correlations between different indicators, both to avoid impact double counting and to reduce the need for data, if possible. Finally, the indicators will be weighed to suggest single scores for environmental and social sustainability, respectively. Weighting is the most complex part of this study, involving different approaches and inherently value-based assessments. Building on established weighting methods, such as the PEF weighting scheme^(41)^, and approaches like distance to target, planetary boundaries, expert panel^(42,43)^ and willingness to pay^(23)^, we will further develop and adapt a system suitable for assessing food sustainability within NewTools. To support transparent and structured prioritisation, we will also explore criteria such as RACER (Relevant, Acceptable, Credible, Easy, and Robust)^(44,45)^, which can provide a partially objective basis for justifying weighting decisions^(45,46)^. The framework will be tested during the development process with data representing high-consumption volume products on the Norwegian market. Partners will be involved in the development of the scoring systems through interviews, questionnaires, dialogue meetings, workshops and sharing of data. This collaboration builds on the strong engagement and interest expressed by the partners already during the project development phase, when the need for a more holistic sustainability framework was highlighted as a knowledge gap and strategic priority. We envision a two-way interaction where partners contribute practical insight, data, and value perspectives from different parts of the food value chain, and in return gain access to tools that may support internal decision-making, communication and innovation. However, potential challenges include diverging expectations regarding the framework’s level of detail and usability, as well as differences in data availability, willingness to share sensitive information and views on how social and environmental indicators should be weighted.

### Infrastructure for collaboration and sharing methods and data

One important way of facilitating food system transformations is enabling efficient use of validated data and methods^(47)^. Therefore, a main aim is to provide tools and facilities for cooperation and sharing of data and methods. Starting with the participants in the project, the ultimate goal is to serve and involve a wider audience.

Handling of the scores being developed, and their supporting data, may involve a wide range of data and methods for evaluation, verification, adaptation, contextual assessment and further development. As summary scores can be used for planning, development and evaluation of activities, as indicated in the right-hand box in Figure 1, they may be used as parts of larger tool kits tailored to the specific needs of the respective food system actors. As such, they may be subject to adjustments and refinement over time, relative to specific applications.

To avoid ‘black box’ situations where the data sources, methods and reasons for assessments and decisions are not transparent, quality assurance, dialogue, communication and cooperation are essential. Thus, open versions of the data and methods used in the project are needed, developed through a participatory, consensus-based approach^(48)^. The data, methods and communication tools can be shared through portals, and if none is commonly available, a cloud-based portal will be developed that, over time, may be used by the public, agencies, the government, other projects and professionals working in the field^(49)^.

The infrastructure developed may continue, eventually with other organisations responsible, with small maintenance and operation costs after the end of the project period. If the mode of cooperation catches hold, the basic portal structure and corresponding desktop tools may later be easily integrated into a more comprehensive infrastructure. Still, the realisation of establishing the infrastructure for sharing methods and data requires close collaboration between partners and may face potential risks, such as resistance to share data.

### Identify, co-create and test applications of the scoring systems

An important part of the NewTools project is to explore how the scoring systems can be applied across the food system. To achieve this, a co-creation approach method will be applied^(48)^, involving all interested project partners. Insights from this process will provide a basis for designing up to three pilots that can be scaled up in future spin-off projects. Mixed methods pilot studies will be used to develop applications and test the usefulness among end users (e.g. food producers, retail, consumers, procurement and policy makers).

In practice, partners’ involvement in this activity will occur through a series of workshops. The aims of the workshops are to (1) identify potential application areas for the NewTools scores and (2) use participatory approaches for identifying and selecting pilots and arenas for testing. The pilots will be further developed and conducted in close collaboration with the partners who commit to participate in the testing. Meanwhile, continuous efforts will be made to include the expertise of all partners throughout the process to increase the utility and impact of the pilots and co-creation activities. Transparency through this iterative process will be ensured by the close communication of aims and expectations before, and sharing reports after, each workshop, including the researchers’ considerations of the input received during discussions. In line with the responsible research and innovation approach^(50)^, partners will be invited to collaborate on evaluating the project at the end of the project period.

## Significance and implications

NewTools is a cross-disciplinary project that brings together nutrition, agricultural, marine and environmental sciences and has established cooperation between diet, food production and sustainability experts as well as partners representing different parts of the food system (Table 1). This has the potential to contribute to a shared understanding of our health and environmental challenges and to develop wider support for policy actions and necessary consumer behaviour shifts. If this is successfully handled, NewTools may contribute to knowledge that may be relevant at the national (Norway), regional (Nordic countries), and European levels. Thus, it may contribute to European progress towards a more sustainable food system while at the same time catering for national and Nordic concerns.

A number of challenges are expected within a consortium spanning so many actors. Conflicting interests, goals and expectations among partners may complicate the necessary clarification of what a sustainable diet and food production essentially entails. Furthermore, there may be widely differing assessments, not only of the perceived value of scoring systems and their applications but also of the research process itself and the outcome impact of the research findings.

All partners were involved in the research application process where over-arching global challenges with the food system were described. Furthermore, everyone agreed with Norway’s political goals to support the UN’s Sustainable Development Goals (SDG). However, when these general goals are to be transformed into practical tools, many issues may be resolved in several ways, necessitating challenging discussions about priorities. A number of associated risks were foreseen in the initial discussions, and it is expected that others may emerge through the project period, requiring proactive project management.

While foreseeing conflicting interests when discussing indicators and weighing in the development of scores, project-wide discussions will probably also reveal deeper issues. There might come concerns regarding the scoring systems themselves and the roles that they can and cannot play in transforming the food system. This is due to the inherent complexity of the food system and the risk of unintended consequences if simplified summary scores are used in decision making. This concern might become particularly high if these scores are used for mandatory front-of-pack labelling. Within this complex project landscape, it is expected that different partners will take different roles; researchers will focus on the evidence and science-based project outputs and the data that underpins the scoring systems, whilst non-research partners might take a more practical approach and focus on the potential consequences of the tools being implemented, perhaps maintaining that the overall impact of the results from the project should be thoroughly considered. The balance between a reductionistic approach and a systems approach is highly relevant from the overarching perspective of food system transformation.

## Conclusion

This paper provides an overview of the novel and ambitious approach taken in the NewTools project to develop tools for food system transformation through broad-based collaboration, presenting the objectives, design and approaches applied. The project has the potential to exert an impact on several research fields, including political and social sciences, nutrition, agriculture and sustainability sciences. With the twenty-eight cross-sector partners, the project hopes to create a systemic impulse, a ‘ripple effect’ throughout the food sector, improving nutritional, social and environmental performances. The lessons learned from the collaborative approach where many sectors and different partners are involved may be valuable to instigating similar projects and initiatives in other countries/settings. The indicators and summary scores developed within a systems approach at national and regional level are warranted and will contribute to a field of research important for evidence-informed action and policy development, like pressure for raising standards, supply- and demand-wise and the cooperative process and shared platform for data, methods and knowledge will, making transformations easier.
